# Enhanced antifouling properties of marine antimicrobial peptides by PEGylation

**DOI:** 10.3389/fbioe.2023.1124389

**Published:** 2023-01-26

**Authors:** Tong Lou, Xiuqin Bai, Xiaoyan He, Wencheng Liu, Zongcheng Yang, Ying Yang, Chengqing Yuan

**Affiliations:** ^1^ Reliability Engineering Institute, National Engineering Research Center for Water Transport Safety, Wuhan University of Technology, Wuhan, Hubei, China; ^2^ Hubei Longzhong Laboratory, Xiangyang, Hubei, China; ^3^ School of Pharmacy and Bioengineering, Keele University, Staffordshire, United Kingdom

**Keywords:** marine biofouling, marine-based peptide, PEGylation, aryldiazonium salts, molecular dynamics simulation, aluminium surface

## Abstract

Covalent immobilisation of antimicrobial peptides (AMPs) on underwater surfaces to combat marine biofouling is of great interest as it is an efficient, broad-spectrum and environmentally friendly strategy. Similar to post-translational modifications of natural proteins, artificial modifications of antimicrobial peptides can introduce important impacts on their properties and functions. The present work revealed the enhanced effect of PEGylation on the antifouling properties of marine antimicrobial peptides (LWFYTMWH) through grafting the modified peptides on aluminium surfaces. PEG was coupled to the peptide by solid-phase peptide synthesis, and the PEGylated peptides were bioconjugated to the aluminium surfaces which was pre-treated by aryldiazonium salts to introduce carboxyl groups. The carboxy group has been activated through the reaction with 1-(3-dimethylaminopropyl)-3-ethylcarbodiimide hydrochloride and N-hydroxysuccinimide. The successful modification was confirmed *via* FT-IR and XPS. Interestingly, the PEGylated peptides modified surfaces could kill 90.0% *Escherichia coli* (Gram-negative) and 76.1% *Bacillus sp*. (Gram-positive), and showed better antifouling performance than the original peptides modified surfaces. Furthermore, molecular dynamics simulations showed PEGylation could enhance the ability of peptides to destroy membrane. The PEGylated peptides inserted into the membrane and induced the change in local curvature of membrane, leading to the rupture of membrane. The presence of PEG changed the antimicrobial peptides into more flexible conformations and the high hydrophilicity of PEG hindered the settlement of bacteria. These might be the two main working mechanisms for the increased antifouling efficiency of PEGylated peptides modified surface. This study provided a feasible modification strategy of antimicrobial peptides to enhance their antifouling properties.

## Introduction

Marine biofouling, defined as the settlement of undesirable molecules and organisms on submerged surfaces, causes serious economic, ecological and secure problems (Jin et al., 2022b). The fouling organisms settled on the surface of marine vessels can promote corrosion, increase drag and destroy the hull, resulting in additional fuel consumption and maintenance costs. The original stable structure of offshore platforms can also be destroyed by fouling organisms, leading to shorter life spans and higher costs. Marine fouling is a serious obstacle to the exploitation of marine resources and the development of the shipping industry (Tuck et al., 2022). However, taking the marine environment into consideration, traditional antifouling coatings, which act on both target and non-target organisms, have been banned (Selim et al., 2022). As a result, the next-generation effective and environmentally compatible antifouling strategies should be developed to combat fouling organisms. In the past few decades, many environmentally friendly antifouling technologies have been developed (Selim et al., 2018; He et al., 2020; Jin et al., 2020; Tian et al., 2021). Among them, bioinspired antifouling technologies have become a research hotspot because of high efficiency and environmental compatibility. And the researches about bioinspired antifouling technologies focus on six major strategies (Jin et al., 2022a); micro/nanostructured surfaces (Selim et al., 2019; Selim et al., 2020), natural antifoulants (Lou et al., 2022), mucus-like hydrogels (Melrose, 2019), slippery liquid-infused porous surfaces (He et al., 2021; Yang et al., 2021), dynamic surfaces (Xie et al., 2019), zwitterionic coatings (Zheng et al., 2017).

In addition, marine biofouling is a complex process which increases the difficulty of developing the new generation of antifouling technology. Generally, biofouling consists of four typical stages; the formation of conditioning film, biofilm, soft macrofouling community and hard macrofouling community (Rosenhahn et al., 2010). Among them, biofilm formed by bacteria is a crucial stage, which acts as an adhesive to fix macrofoulers firmly on the submerged surfaces (Jain and Bhosle, 2009; Wang et al., 2022). Therefore, ways to reduce the settlement of bacteria on subaqueous surfaces are strategic to prevent biofouling. Antimicrobial peptides (AMPs), a gift from nature to fight bacteria, are ancient host defence effector molecules. AMPs have broad-spectrum antimicrobial potential to combat both Gram-positive and Gram-negative bacteria even at low concentration. As a matter of course, AMPs are frequently studied in marine antifouling field. Cao et al. (Cao et al., 2020) used dopamine to graft the Magainin Ⅱ onto the 304 stainless steel surfaces, and the modified surfaces reduced 98.07% adhesion rate of *Vibrio natriegens*. Beyer et al. (2020a); Beyer et al. (2020b); Beyer et al. (2021) prepared a series of peptide self-assembled monolayers (SAMs) on gold-coated Nexterion B slides to combat marine biofouling. The peptide SAMs exhibited excellent antifouling properties to inhibit the settlement of *Navicula perminuta* (up to 97%) and *Cobetia marina* (up to 50%).

Similar to post-translational modifications of natural proteins, artificial modifications of AMPs can have important impacts on their properties and functions. PEG is widely used for the benefits it provides when conjugated to peptides, such as improvement in solubility, stability of proteolysis, and reduction in rapid clearing by the kidney and liver (Hamley, 2014; Chen et al., 2019). PEGylation has also been used to enhance the antimicrobial properties of AMPs. Ju et al. (2020) designed a PEGylated peptide, which can easily pass through extracellular polymeric substance (EPS) *via* the water-filled channels, showing much higher antibacterial efficiency than original peptide (72.70% *versus* 15.24%). Ortiz-Gomez et al. (2020) coupled PEG to Maximin H5, and the conjugate had increased antimicrobial properties than Maximin H5, due to the new function of preventing biofilm formation and eradicating biofilm for *Pseudomonas aeruginosa* and *Escherichia coli*. In addition, it has been reported that PEGylation could increase the stability of the antimicrobial peptide, M33, to *Pseudomonas aeruginosa* elastase, which is a virulence factor to destroy peptides, resulting in a doubling of antimicrobial properties over the original peptide (Falciani et al., 2014). Although PEGylated peptides in the free state have been extensively studied, little is known about the marine antifouling properties of PEGylated peptides immobilized on the surface. Furthermore, there are few studies about the antifouling mechanism of PEGylated peptides.

Herein, this work investigated the effect of PEGylation on antifouling properties of AMPs to make up for current vacancy of relative researches and to provide a feasible modification strategy of AMPs to enhance their antifouling properties. Based on the previous research (Lou et al., 2022), the marine-based peptides (LWFYTMWH) was modified by PEGylation *via* Fmoc-protected solid-phase peptide synthesis method. Then, PEGylated peptides were bioconjugated to aluminium surfaces (#5083) by dehydration condensation reaction between carboxyl groups on aluminium surfaces and amino groups on peptides under the activation of EDC and NHS. Both *Bacillus* sp. (Gram-positive) and *Escherichia coli* (Gram-negative) were employed to evaluate the antifouling performance of as-prepared specimens. The interactions between phospholipid membranes and PEGylated peptides were investigated using molecular dynamics simulations to provide further insight into the antifouling mechanism of PEGylated AMPs under the molecular level.

## Materials and methods

### Chemicals and materials

Aluminium alloy discs (#5083, Φ 10 mm) were used as substrate. Tert-butyl nitrite (t-BuONO, 90%), 4-aminobenzoic acid (99%), fluoroboric acid solution (HBF_4_, >40%), 1-(3-dimethylaminopropyl)-3-ethylcarbodiimide hydrochloride (EDC, 98.5%), N-hydroxysuccinimide (NHS, 98%), were all obtained from Macklin and utilized without further treatment. 4-carboxybenzenediazonium tetrafluoroborate was prepared by the reaction between 4-aminobenzoic acid and t-BuONO, as reported in previous work (Lou et al., 2022). 2216E agar, 2216E liquid medium, LB agar and LB broth were purchased from Qingdao Hope Bio-Technology Co., Ltd. and used as described in the instructions to culture bacteria. Artificial seawater (ASTM D1141-98), and utensils utilized for biofouling tests were autoclaved before use.

### PEGylated peptide synthesis

The derived peptide (H_2_N-PEG2-LWFYTMWH-COOH) was prepared by standard Fmoc-protected solid-phase peptide synthesis (Loo et al., 2021). The rink amide resin (1 equiv.) was swelled in dichloromethane (DCM) solution for 5 min. After that, the protecting group, Fomc, was deprotected in N, N-dimethylformamide (DMF) solution with 20% piperidine for 10 min. According to sequence, the Fmoc-His (Trt)-OH was coupled to resin in the presence of organic base (DIEA, 8 equiv.) and condensation reagent (PyBOP, 4 equiv). The steps of deprotection and coupling were repeated until the target peptide was synthesized. The H_2_N-PEG2-COOH was also coupled by the same method. During the synthesis process, the efficiency of deprotection and coupling was analysed by the Kaiser test. The resin and protecting groups were finally cleaved by the trifluoroacetic acid (TFA) solution with 2.5% H_2_O and 2.5% triisopropylsilane. The obtained PEGylated peptide was purified and lyophilized, then stored at −20°C until used.

### Surface modification by PEGylated peptides

**SCHEME 1:**
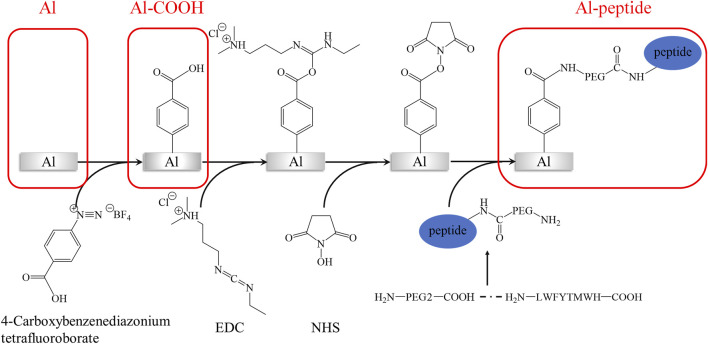
The main generation process of PEGylated peptide-modified aluminium alloy antifouling surfaces.

The main generation process of peptide-modified aluminium alloy (Al) antifouling surfaces was shown in Scheme 1. Al discs were polished by sandpapers (800#, 1000#, and 2000#) and alumina slurry (0.05 μm), and sequentially ultrasonicated in acetone, alcohol, deionized water. The specimens were recorded as Al. After etched by 0.1 M sodium hydroxide solution for 5 min, Al discs were immersed in a 10 mM 4-carboxybenzenediazonium tetrafluoroborate solution with 0.01M HBF_4_ for 8 h. The specimens were recorded as Al-COOH. And then, a solution containing 0.1 M NHS and 0.4 M EDC was utilized to activate the carboxyl groups on the Al discs for 4 h. Finally, Al discs were submerged in 0.1 M acetic acid solution with 0.5 μg/ml PEGylated peptide for 24 h at room temperature. The specimens were recorded as Al-peptide. Notes that deionized water was used in each step to rinsing thoroughly the Al discs to remove unreacted reagents.

### Characterization of the specimens

Fourier transform-infrared spectroscopy (FT-IR; AVATAR 360, Nicolet Instrument Corp., United States) was used to analyse the different functional groups on the surfaces of Al, Al-COOH and Al-peptide specimens, with a resolution of 4 cm^−1^ and eight co-added scans. The X-ray photoelectron spectra were collected by the X-ray photoelectron spectroscopy (XPS; ESCALAB 250Xi, Thermo Fisher Scientific Inc., United States), with Al-Kα (1486.6 eV) as the radiation resource.

### Biofouling testing

The *Escherichia coli* (*E. coli*, ATCC 25922) and *Bacillus sp*. (MCCC 1A00791) were employed as the model bacterial strains to determine the antifouling performance of specimens. The *E. coli* strains were streaked onto LB agar plates and cultured at 30°C to obtain single colony. And then the single colony was transferred to LB broth. After growing in an incubator (30°C, 120 rpm, 24 h), the bacteria were diluted by LB broth until the concentration of *E. coli* suspension was 1 × 10^5^/ml. Changing the bacterial medium to 2216E agar and 2216E liquid medium, the *Bacillus sp*. suspension (1 × 10^5^/mL) was prepared using the same way.

The antibacterial capability of specimens was tested according to GB/T 21866-2008. Briefly, 50 μl bacterial suspension was spread on each surface of specimens, and a sterile polyethylene film (1 × 1 cm) covered each surface to ensure an even distribution of bacteria. After cultured at 30°C in the dark for 24 h, 50 ml seawater was used to completely elute the bacteria on surface and film. Subsequently, 50 μl eluent was pipetted onto agar plate and cultured for 24 h to count the viable bacteria number on each specimen. The antibacterial rate of Al-peptide specimens was obtained using Eq. 1.

$$A\;\%=\left[\left(B-C\right)/B\right]\times 100\%$$ (1)

Where, A% represented the antibacterial rate, B and C meant the viable bacteria number on the Al specimens and the Al-peptide specimens, respectively.

The morphological characteristics of *E. coli* and *Bacillus sp*. on surfaces of three different specimens were deeply analysed by field emission scanning electron microscopy (FESEM; SIRION, FEI Co., United States). Briefly, three different specimens were fully immersed by bacterial suspension and incubated in an incubator with rotation (30°C, 120 rpm, 48 h). After rinsed by seawater, specimens were immersed in 2.5% glutaraldehyde solution more than 4 h to fix the bacteria. Before observed using FESEM, the specimens were sequentially dehydrated through 25%, 50%, 75%, 90%, and 100% ethanol solutions, and dried in a vacuum dryer (37°C, 24 h).

### Molecular dynamics simulations

The simulations of the interaction between cell membrane and peptides with long-term and large-scale have been undertaken to gain deep insight into the interactions between membranes and peptides. Membranes are essential components of cell, containing a large variety of lipids, proteins and carbohydrates, which makes it difficult to carry out studies on real cell membranes (Marrink et al., 2019). Typically, phospholipid bilayers formed by single- or multi-component phospholipid molecules are used to reasonably simplify cell membranes. The membranes of Gram-negative bacteria commonly consist of two main types of lipids, palmitoyloleoyl phosphatidylglycerol (POPG) and palmitoyloleoyl phosphatidylethanolamine (POPE) (Dowhan, 1997). Therefore, a bacterial-mimetic cell membrane with a 1:3 ratio of POPG to POPE lipids was simply used to investigate the effect of PEGylated peptide on cell membrane (Fang et al., 2018). Fortunately, coarse-grained molecular dynamics (CG MD) simulations can probe length and time scales beyond the scope of all atom molecular dynamics simulations. Among all, Martini force field is widely employed to simulate the interactions between lipid bilayers and peptides (Monticelli et al., 2008; Ermakova and Zuev, 2017; Frallicciardi et al., 2022).

**FIGURE 1:**
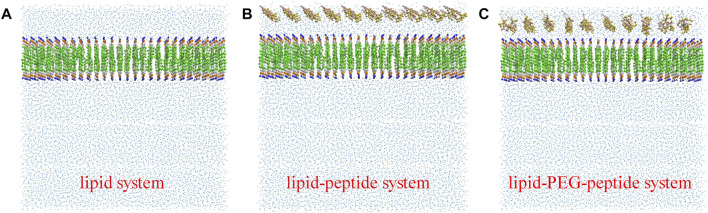
The coarse-grained models of lipid system **(A)**, lipid-peptide system **(B)** and lipid-PEG-peptide system **(C)**.

**FIGURE 2:**
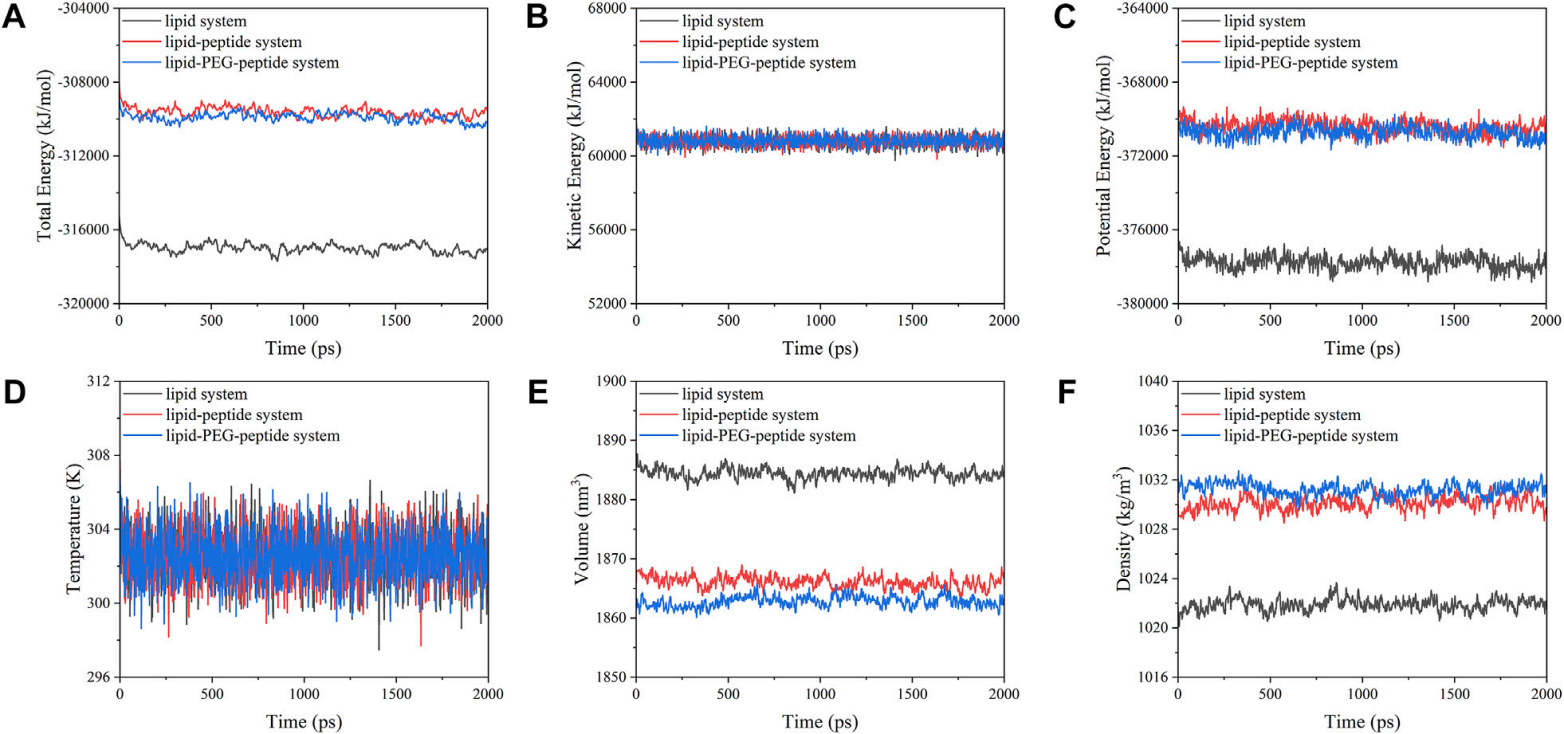
Evolution of total energies **(A)**, kinetic energies **(B)**, potential energies **(C)**, temperatures **(D)**, volumes **(E)** and densities **(F)** of the systems with time.

In this work, the CG MD was relied on the latest version, Martini three force field (Souza et al., 2021; Alessandri et al., 2022) and run using GROMACS package (2019.6) with GPU acceleration. The lipid system was generated by the tool Insane (Wassenaar et al., 2015) to construct a 20 × 5 × 20 nm^3^ box, containing 78 POPG lipid molecules and 234 POPE lipid molecules (Figure 1A). And the charge of the system was neutralized with 0.15 M ions (259 Na^+^, 181 Cl^−^). The CG models of original peptide and PEGylated peptide were generated by the tool Martinize2 according to the protocol for simulations of PEGylated proteins with Martini three force field. The XY plane was uniformly divided into a 10 × 2 grid, and 20 original peptides or 20 PEGylated peptides were regularly inserted into the lipid system to construct the lipid-peptide system (Figure 1B) or lipid-PEG-peptide system (Figure 1C). All N-terminals of peptides were frozen in the XY plane of Z = 0 to mimic the immobilized state. Firstly, the systems were energy minimized to eliminate the unreasonable spatial structure. And due to the large forces involved it was necessary to run a few equilibrium simulations in the NPT ensemble at 303K using a short time step (1, 5, and 10 fs). Then, simulations were performed with standard time step of 0.02 ps for 2 ns to evaluate the stability of the systems. The total energies, kinetic energies, potential energies, temperatures, volumes and densities of the systems reached stable status, as show in Figure 2. Finally, the systems were conducted production simulations for 2000 ns with time step of 0.02 ps, and the trajectories were recorded every 0.2 ns. A v-rescale thermostat (Bussi et al., 2007) was used for temperature coupling at 303 K, separately for different groups (lipids/solvent for lipid system, lipids/solvent/peptide for lipid-peptide system, lipids/solvent/PEGylated peptide for lipid-PEG-peptide system). The simulation pressure was coupled to a Parrinello–Rahman barostat at 1 bar independently for *Z*-direction and X/Y directions. The MembraneCurvature tool and LiPyphilic tool (Smith and Lorenz, 2021) on MDAnalysis library (Michaud-Agrawal et al., 2011) were applied to analysis last 20 ns of trajectories with 100 frames.

## Results and discussion

### Characterizations of as-prepared specimens

**FIGURE 3:**
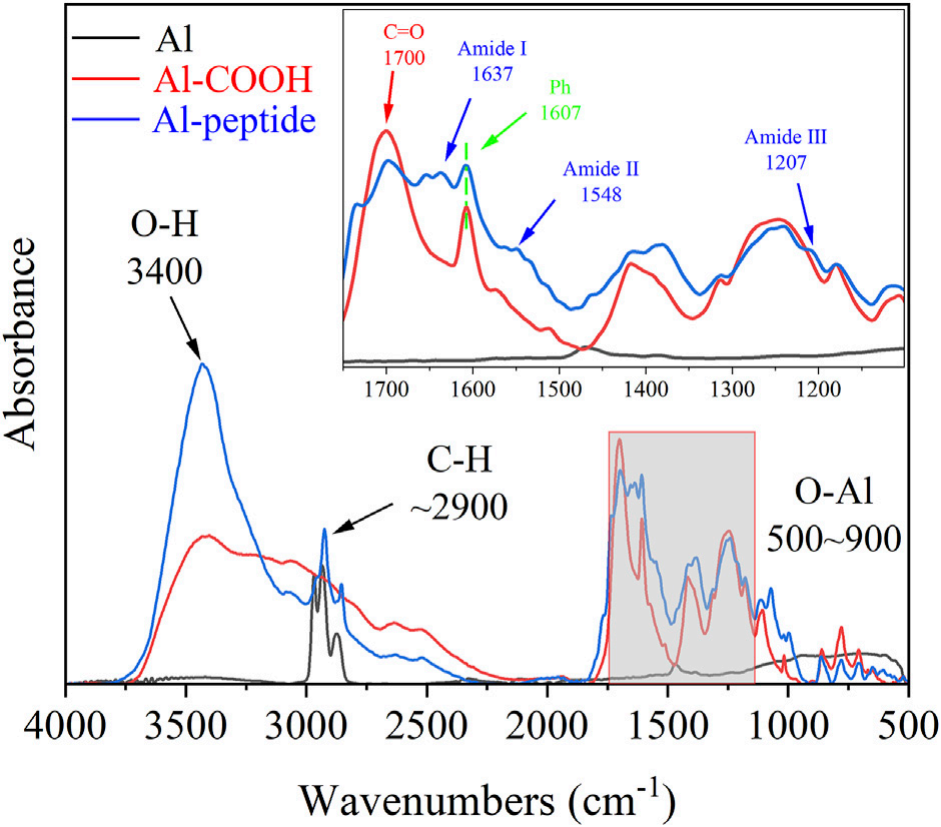
FT-IR spectra of Al specimens, Al-COOH specimens, and Al-peptide specimens.

The chemical compositions of as-prepared specimens were characterized using FT-IR and XPS analyses. The FT-IR spectra showed notable difference between the three specimens, Al specimens, Al-COOH specimens and Al-peptide specimens (Figure 3). There were only two regions with distinct peaks on the spectrum of Al specimens, the peaks between 900 and 500 cm^−1^ caused by the vibration of Al-O groups (He et al., 2019), and the peaks at 2968, 2933, and 2873 cm^−1^ attributed to stretching vibration of alkyl groups. And, three new typical peaks presented on Al-COOH specimens to imply the existence of benzene ring and carboxyl groups. A peak appeared at 1607 cm^−1^ because of the vibration of aromatic ring, besides, a distinct peak at 1700 cm^−1^ and a broad peak at 3400 cm^−1^ were respectively contributed to the stretching vibration of C=O and O-H (Berisha et al., 2016), which were part of carboxyl groups. Except the peaks mentioned above, the vibration of protein backbone led to three characteristic peaks on the spectrum of Al-peptide specimens, amide Ⅰ at 1637 cm^−1^, amide Ⅱ at 1548 cm^−1^, and amide Ⅲ at 1207 cm^−1^ (Govindan et al., 2021; Lan et al., 2021). The results of FT-IR spectra revealed the PEGylated peptides had been modified successfully on Al discs.

The contents of oxygen, aluminium, carbon, nitrogen, sulphur on different as-prepared specimens obtained from semi-quantitative analysis of XPS detection also indicated the PEGylated peptides were successfully grafted onto Al-peptide specimens (Table 1). After reaction with diazonium salt, the content of oxygen reduced from 49.73 at% to 20.98 at%, and the aluminium could not be detected on Al-COOH specimens. Moreover, the content of carbon and nitrogen significantly increased from 26.49 at% to 74.26 at% and from 1.15 at% to 4.76 at%, respectively. All of these indicated that the Al substrates were covered by films containing carboxyl groups. Importantly, the appearance of sulphur, as the distinctive element of peptide, was direct evidence of the peptide film on Al-peptide specimens. The change of nitrogen content was also consistent with this conclusion.

**TABLE 1:** XPS semi-quantitative analysis of different specimens.

Specimen types	O (at%)	Al (at%)	C (at%)	N (at%)	S (at%)
Al specimens	49.73	22.64	26.49	1.15	0
Al-COOH specimens	20.98	0	74.26	4.76	0
Al-peptide specimens	19.97	0	70.58	8.97	0.49

**FIGURE 4:**
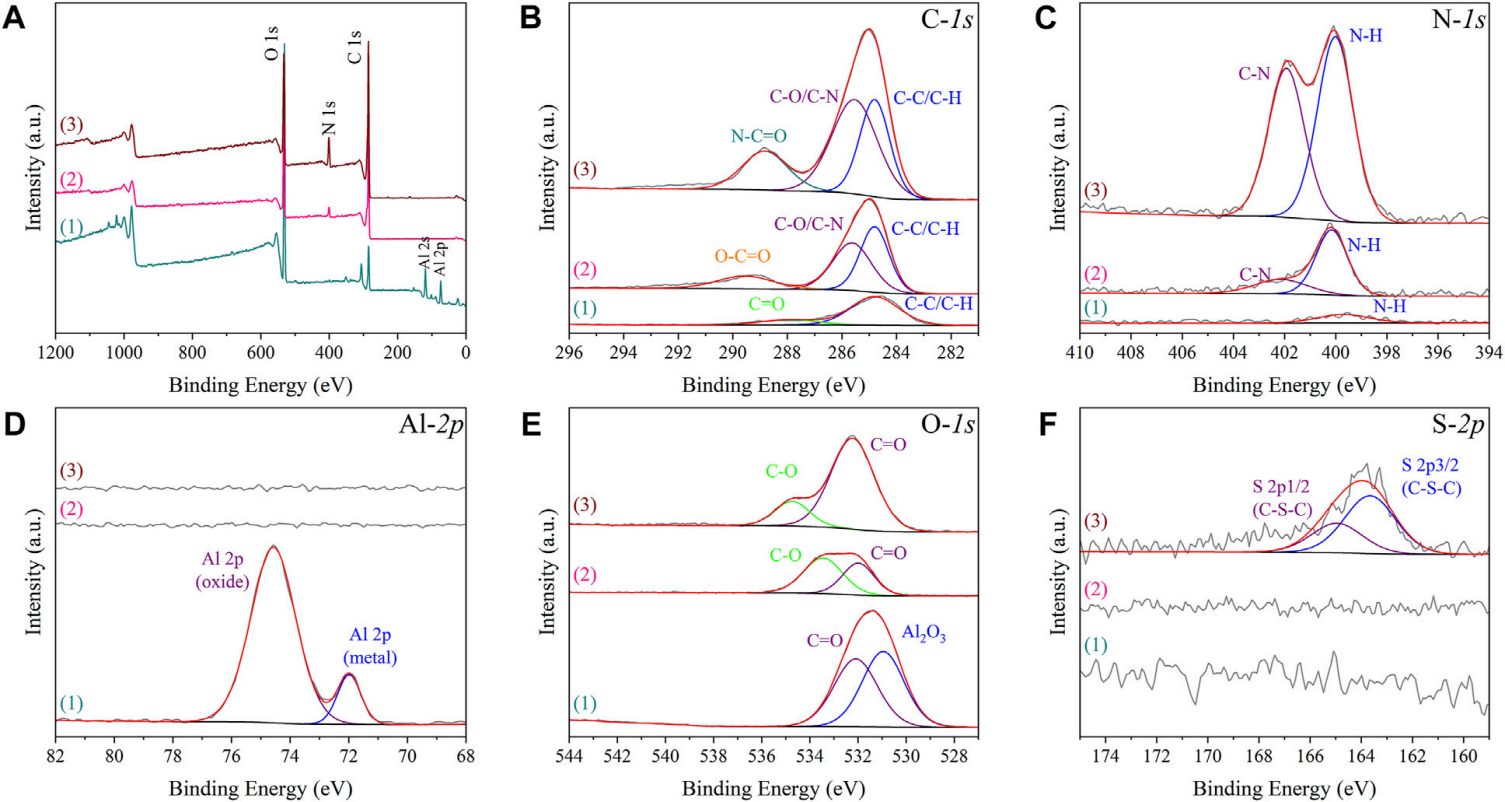
XPS spectra of Al specimens (1), Al-COOH specimens (2), and Al-peptide specimens (3) with XPS survey spectrum **(A)** and C-*1s*, N-*1s*, Al-*2p*, O-*1s* and S-*2p* spectra [**(B–F)**, respectively].

Further analysis of XPS spectra provided compelling evidence of the successful preparation of Al-COOH specimens and Al-peptide specimens (Figure 4). The peaks of Al_2_O_3_ on both high-resolution Al-*2p* and O-*1s* spectra could not be detected on Al-COOH specimens, due to the coverage of films containing carboxyl groups. Meanwhile, the high-resolution spectra of C-*1s* showed the same conclusion. The two peaks at 284.80 eV (C-C/C-H) and 287.52 eV (C=O) on Al specimens were caused by the adsorption of external contaminated carbon (Fang et al., 2020). However, except the peaks at 284.8 eV (C-C/C-H) and 289.54 eV (O-C=O), an additional peak appeared on Al-COOH specimens at 285.63 eV (C-O/C-N), which proved the existence of carboxyl groups (He et al., 2018). Furthermore, accompanied by the reaction between amino groups and carboxyl groups, the carbon peak shifted from 289.54 eV (O-C=O) to 288.82 eV (N-C=O) (Cao et al., 2018). Noteworthy, there was a pair of characteristic S-*2p* peaks at 163.66 eV and 164.96 eV (C-S-C) on Al-peptide specimens, but Al-COOH specimens not, which proved the existence of PEGylated peptide (Wirde et al., 1999; Lou et al., 2021).

### Antifouling performance of as-prepared specimens

**FIGURE 5:**
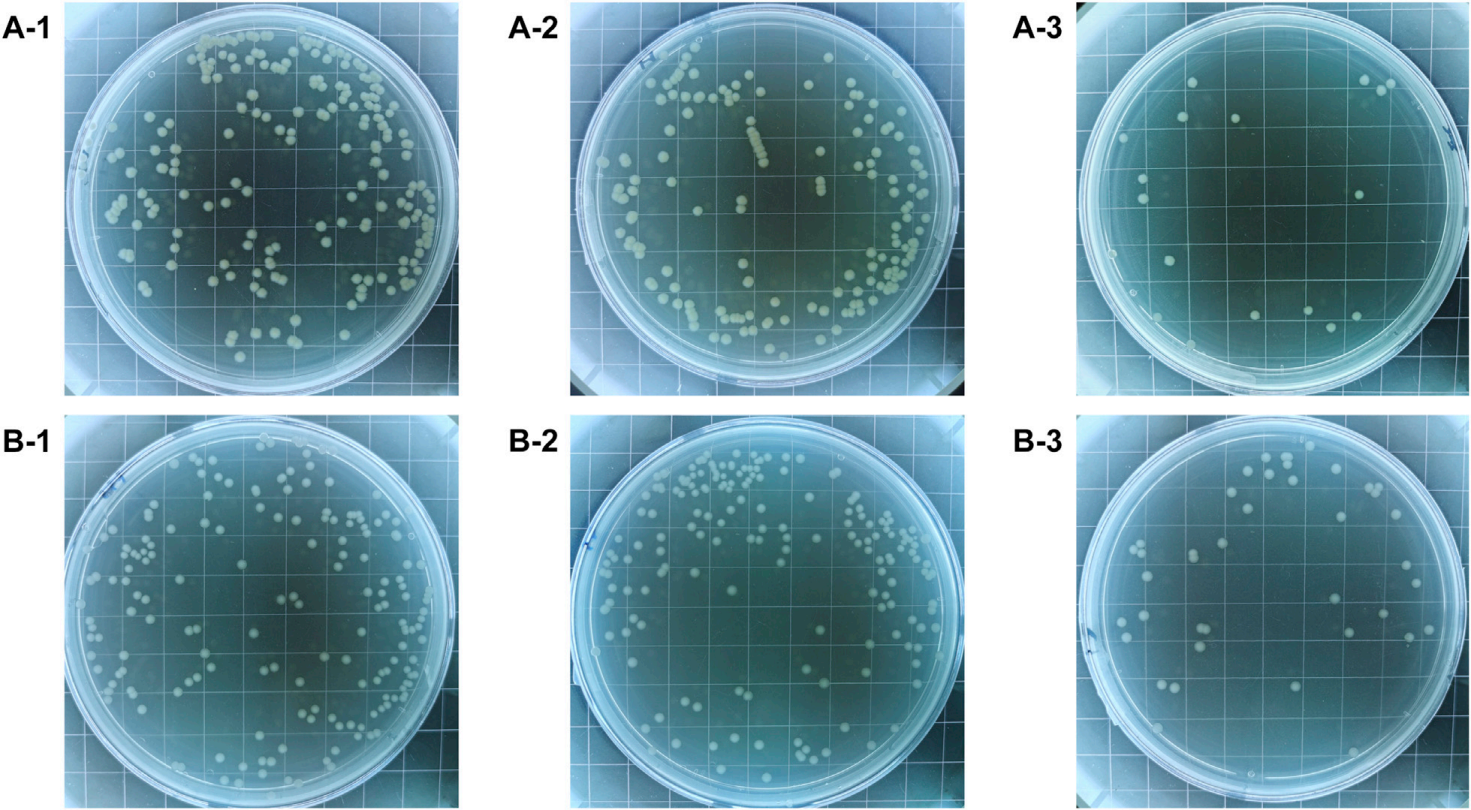
The colony-forming units of *E. coli* **(A)** and *Bacillus* sp. **(B)** after incubated on the Al specimens (1), Al-COOH specimens (2), and Al-peptide specimens (3).

**FIGURE 6:**
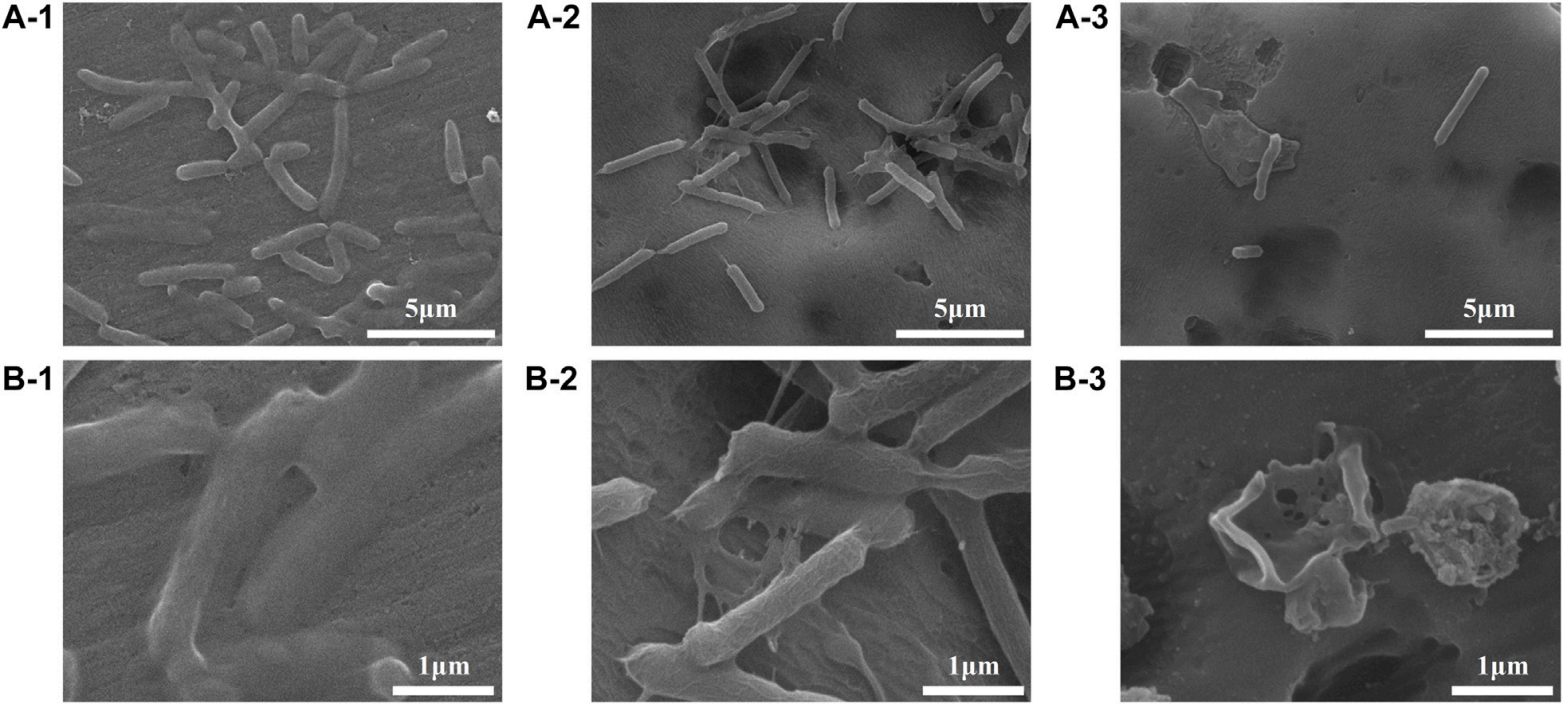
FESEM images showing the adhesion behaviours of *E. coli* on the Al specimens (1), Al-COOH specimens (2), and Al-peptide specimens (3) with 5000 × **(A)** and ×20,000 magnification **(B)**.

**FIGURE 7:**
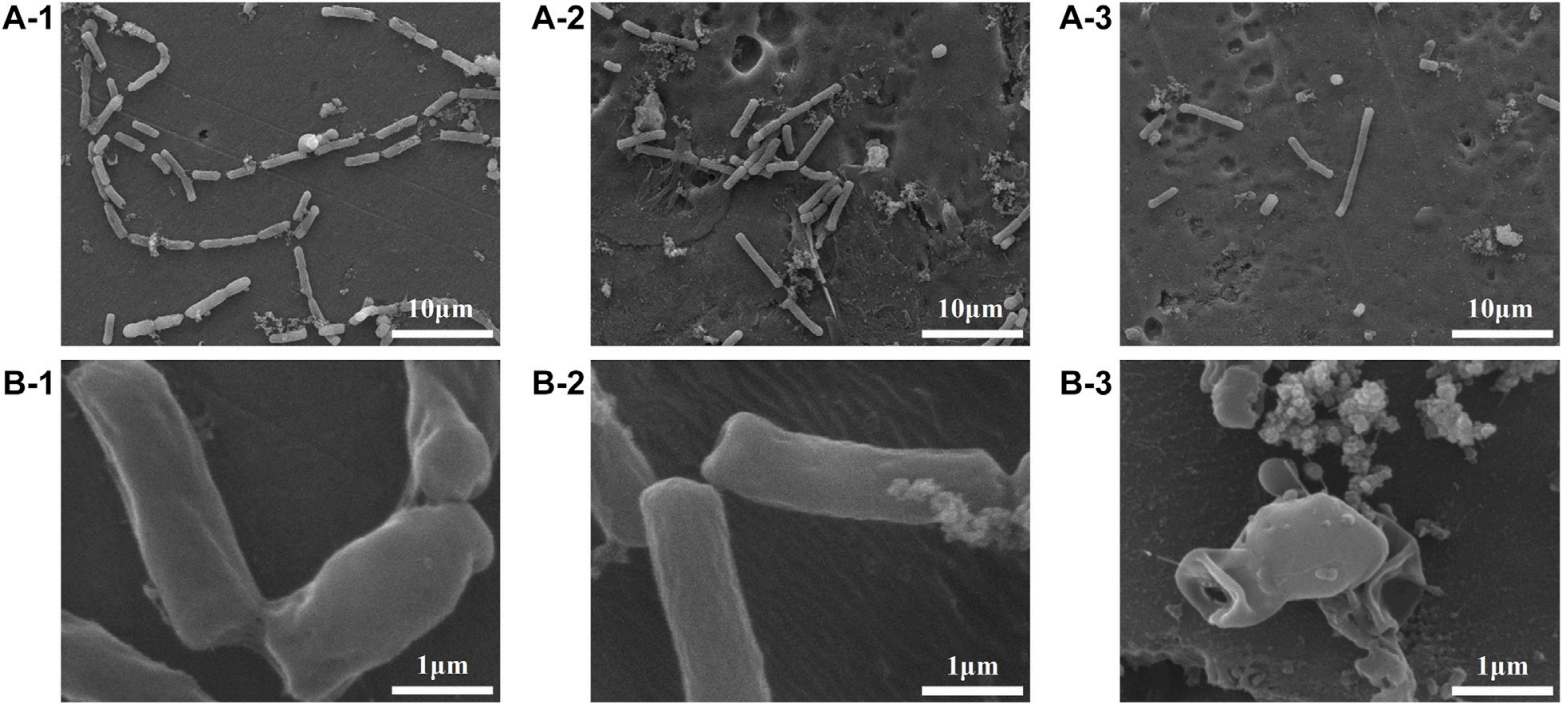
FESEM images showing the adhesion behaviours of *Bacillus sp*. on the Al specimens (1), Al-COOH specimens (2), and Al-peptide specimens (3) with ×2000 **(A)** and ×20,000 magnification **(B)**.

The colony-forming units on three difference specimens showed good antibacterial properties of Al-peptide specimens against both *E. coli* and *Bacillus sp*. (Figure 5). The viable bacteria number of *E. coli* or *Bacillus sp*. on Al-peptide specimens was the least among the three specimens, 90.0% and 76.1% less than viable bacteria on Al specimens, respectively. Comparing with the original peptide we previously reported (78.8% to *E. coli* and 55.4% to *Bacillus sp*.) (Lou et al., 2022), PEGylated peptide modified specimens showed better antibacterial properties with enhanced antifouling properties of peptides. According to morphological characteristics of bacteria on specimens (Figure 6 and Figure 7), the PEGylated peptides immobilized on surfaces killed the bacteria by forming holes in the cell membranes to prevent the attachment of bacteria, leading to a remarkable reduction in the number of viable bacteria attached to Al-peptide specimens, the same strategy as the original peptide to prevent biofouling. PEGylation would not change the antibacterial mode of peptides, the presence of PEG allowed peptides to change their conformations more flexibly and to move laterally more easily, which were required for antibacterial action (Gabriel et al., 2006; Costa et al., 2014; Nie et al., 2017). In addition, the hydrophilicity of PEG could hinder the settlement of bacteria, might be another reason for the increased antifouling efficiency of PEGylated peptides modified surface.

### Simulation of the antibacterial mechanisms of the peptides

**FIGURE 8:**
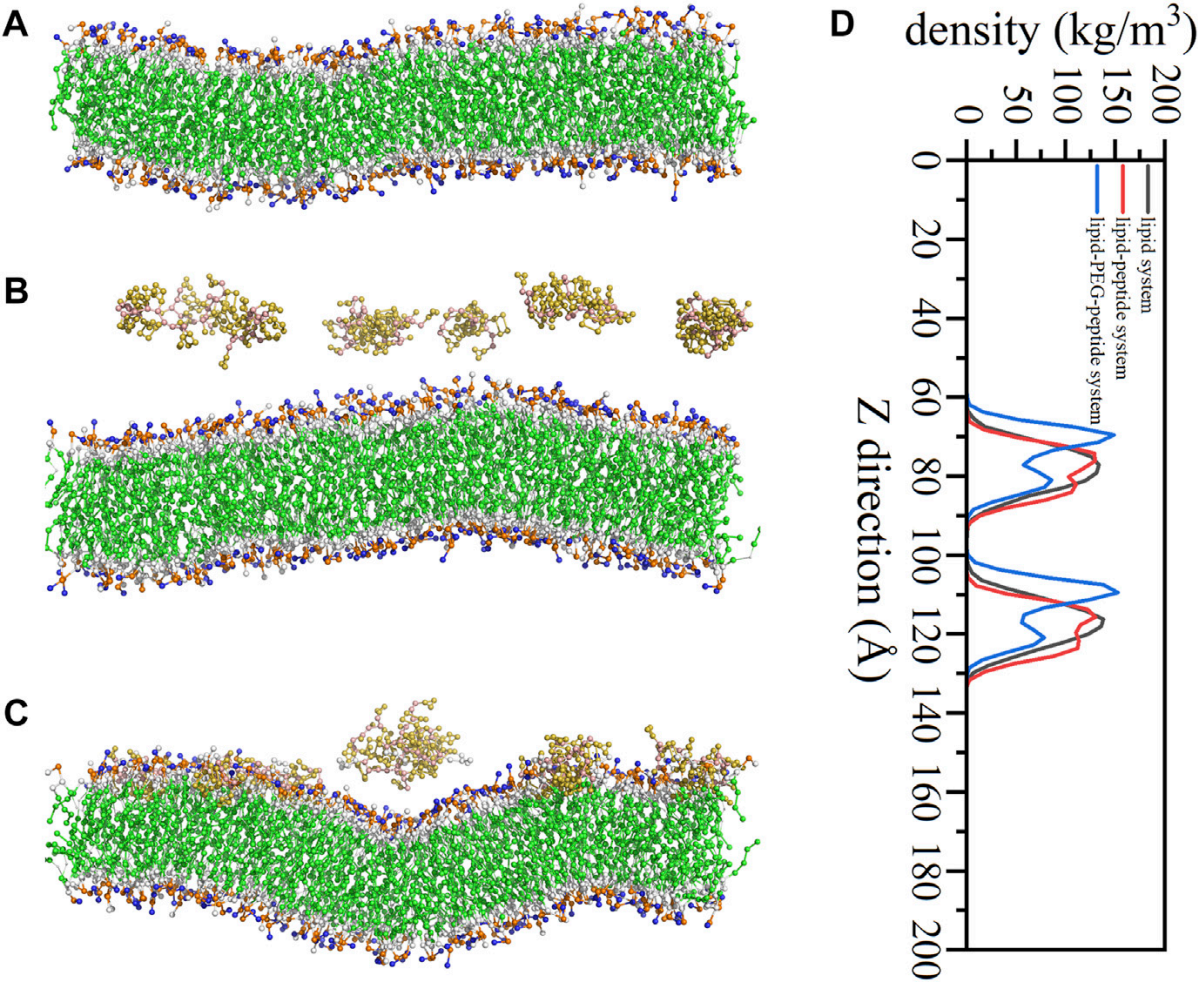
Snapshots of lipid system **(A)**, lipid-peptide system **(B)** and lipid-PEG-peptide system **(C)** at 2000 ns, for clarity, only peptides and lipid bilayers were shown; the concentrations of phosphate groups along the *Z* direction **(D)**.

**FIGURE 9:**
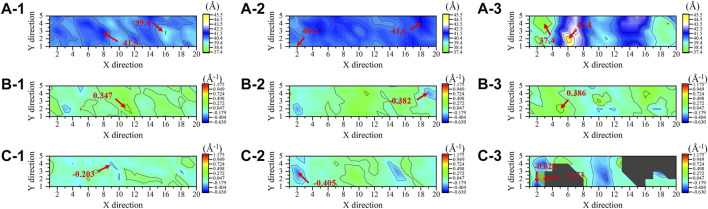
The distances between the phosphate groups in the lower and upper layers **(A)**, the mean curvature of lower layers **(B)** and upper layers **(C)** in lipid system (1), lipid-peptide system (2) and lipid-PEG-peptide system (3).

Molecular dynamics simulations further exposed the disruption of cell membranes by PEGylated peptides to explain the bacterial characteristics observed by FESEM. Different from the lipid system and lipid-peptide system, in the lipid-PEG-peptide system, the bilayer moved towards the edge as attracted by the fixed PEGylated peptides. The trajectories were centered in order to better compare the difference between three systems. It was revealed that the relatively regular bilayer in lipid system was inserted by PEGylated peptides, resulting in significant structural changes (Figure 8), especially the upper layer in direct contact with the PEGylated peptides. However, during the simulation duration, the original peptides did not show the same phenomenon. To further understand the membrane deformation, phosphate groups (PO4 beads) were analysed to represent the bilayers. The distribution of PO4 beads in the lower and upper layers could reflect the important information of membrane, such as thickness and curvature. Based on concentrations of PO4 beads along the Z coordinate (Figure 8D), it was found that the PO4 beads in three systems were mainly concentrated between 60–90 Å and 100–130 Å, and the thickness of membrane was around 40 Å. However, as the bilayer was induced to bend by the peptides, two peaks were formed in both lipid-peptide system and lipid-PEG-peptide system. To investigate the different regions of membranes, the XY plane was divided into a 20 × 5 grid. It was confirmed that the average distances between the PO4 beads in same grid of lower and upper layers were 40.4 ± 0.4 Å, 40.8 ± 0.3 Å and 40.4 ± 1.5 Å in lipid system, lipid-peptide system and lipid-PEG-peptide system, respectively (Figure 9A). However, after receiving induction by the PEGylated peptides, the maximum distance increased from 41.5 Å to 45.4 Å and the minimum distance decreased from 39.4 Å to 37.4 Å, which indicated that the membrane became more dispersed. Although the membrane did not rupture significantly during the simulation duration, the change in local curvature was a clear sign of rupture (Figures 9B, C). The maximum curvatures of lower layer and upper layer in lipid system was 0.347 Å^-1^ and −0.203 Å^-1^ (Figure 9C-1). And in lipid-peptide system, the membrane was slightly more curved (Figure 9C-2). After being inserted by the PEGylated peptides, the curvature of the lower layer was not greatly affected, but the maximum curvature of the upper layer increased significantly to 1.173 Å^-1^ (Figure 9C–3). Even because the spatial positions were occupied by the PEGylated peptides, many regions were devoid of PO4 beads, as shown in the black region of Figure 9C–3. In order to characterize the orientation of lipid molecules in terms of the membrane normal, the (second-rank) order parameters P_2_ were calculated by Eq. 2.

$$P_{2}=\frac{1}{2}\;\left(3\cos^{2}<\theta >-1\right)$$ (2)

**FIGURE 10:**
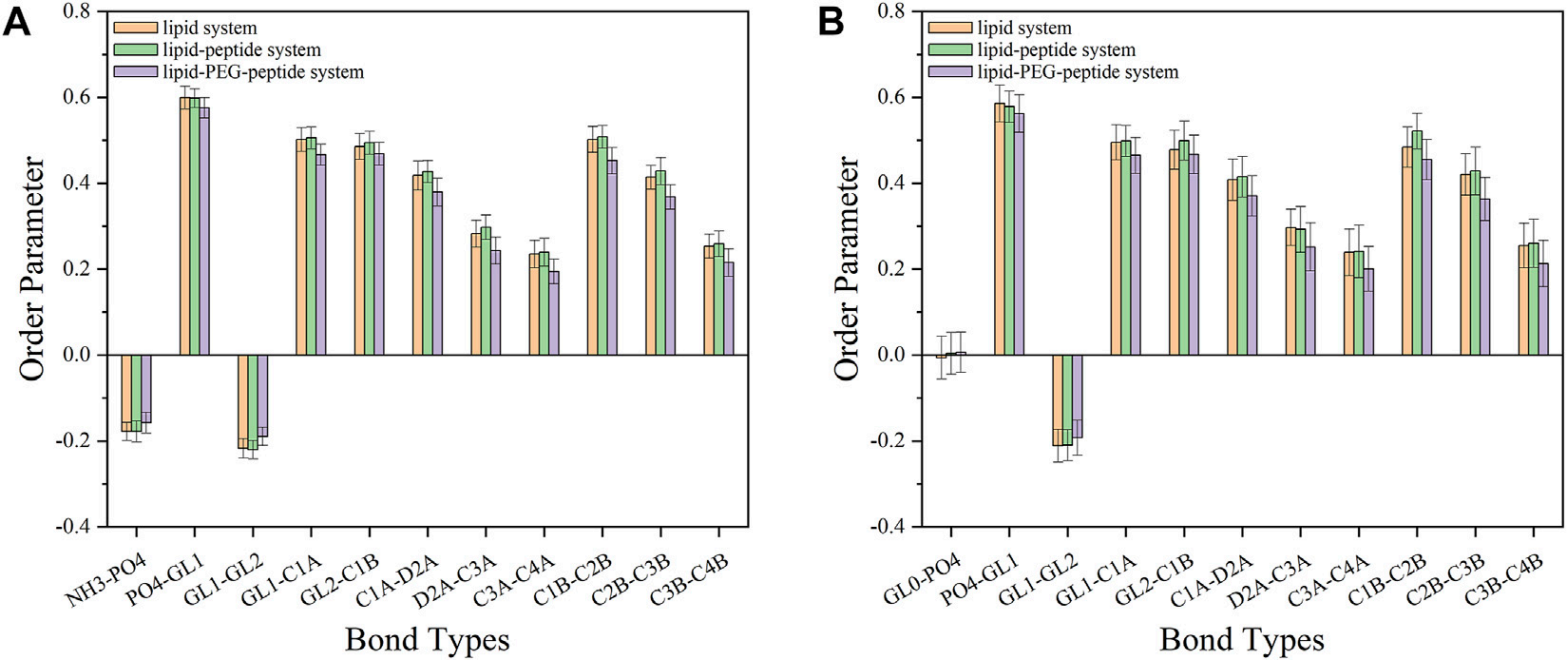
The order parameters of different bonds on POPE lipids **(A)** and POPG lipids **(B)** in lipid system, lipid-peptide system and lipid-PEG-peptide system.

Here, *θ* was the time-dependent angle between the bond and the membrane normal (*Z*-axis). The value of P_2_ ranges from 1 (alignment with the normal) to 0 (random orientation) to −0.5 (perpendicular to the normal). The order parameters of the bonds in the lipid-PEG-peptide system were almost closer to 0 than the corresponding bonds in the other two systems, which meant that the bond orientation in the lipid-PEG-peptide system was more random (Figure 10). According to the simulation results, the original peptides fixed on the surface could only change the membrane slightly more curved during the simulated duration. But, the PEGylated peptides inserted into the lipid bilayer, disrupted the ordered arrangement of the membrane and caused excessive local curvature, eventually leading to the rupture of membrane, which could be seen in the FESEM images. The PEGylation enhanced the ability of peptides to destroy membrane, resulting in increased antifouling performance.

## Conclusion

This study provided a feasible modification strategy of antimicrobial peptides to enhance their antifouling properties by PEGylation. This PEGylated peptide was synthesised by solid-phase peptide synthesis method and then immobilized on aluminium alloy surface by a targeted grafting strategy of amino and carboxyl condensation. The PEGylated peptides modified surface could kill 90.0% *E. coli* and 76.1% *Bacillus sp*., and showed better antifouling performance than the original peptides modified surfaces (78.8% to *E. coli* and 55.4% to *Bacillus sp*.). According to the coarse-grained molecular dynamics simulation results of the three systems (lipid system, lipid-peptide system and lipid-PEG-peptide system), the PEGylation could enhance the ability of peptides to destroy membrane, resulting in enhanced antifouling properties of PEGylated peptides. Different from the original peptides, the PEGylated peptides inserted into the lipid bilayers, disrupted the ordered arrangement of the membrane and caused excessive local curvature, eventually leading to the rupture of the membrane, which could be proved by SEM images. We postulate that, more flexible conformations and stronger lateral movement were the basic reasons for the increased antifouling ability of PEGylated peptides.

## Data Availability

The raw data supporting the conclusion of this article will be made available by the authors, without undue reservation.
